# Feasibility and Tolerability of Ketogenic Interventions in Amyotrophic Lateral Sclerosis—A Dose-Finding Case Series

**DOI:** 10.3390/nu18101628

**Published:** 2026-05-21

**Authors:** Christine Herrmann, Samantha Satari, Andrea Weber, Tanja Ruschitzka, Luisa Jagodzinski, Zeynep Elmas, Felicitas Becker, Lars Richter, Maximilian Wiesenfarth, Sebastian Michels, Jochen H. Weishaupt, Joachim Schuster, Johannes Dorst

**Affiliations:** 1 Department of Neurology, University of Ulm, 89081 Ulm, Germany; christine.herrmann@uni-ulm.de (C.H.); samantha.satari@uni-ulm.de (S.S.); andrea.weber@uni-ulm.de (A.W.); tanja.ruschitzka@uniklinik-ulm.de (T.R.); luisa.jagodzinski@uniklinik-ulm.de (L.J.); zeynep.elmas@uniklinik-ulm.de (Z.E.); felicitas.becker@uni-ulm.de (F.B.); lars.richter@uni-ulm.de (L.R.); maximilian.wiesenfarth@uni-ulm.de (M.W.); sebastian.michels@uni-ulm.de (S.M.); jochen.weishaupt@uni-ulm.de (J.H.W.); joachim.schuster@uni-ulm.de (J.S.); 2 German Center for Neurodegenerative Diseases (DZNE) e. V., 89081 Ulm, Germany

**Keywords:** amyotrophic lateral sclerosis, β-hydroxybutyrate, ketone esters, ketone salts, ketogenic diet

## Abstract

**Background/Objectives:** Weight loss and hypermetabolism are negative prognostic factors in amyotrophic lateral sclerosis (ALS). Ketone bodies (β-hydroxybutyrate, βHB) as high-energy substrates may compensate for this energy deficit, since a ketogenic diet (KD) has been shown to increase survival and stabilize body weight in the *SOD1* mouse model. In this case series, we tested exogenous ketone salts (KS), ketone esters (KE), and a KD, in patients with ALS and in healthy subjects to identify novel therapeutic interventions for subsequent clinical studies. **Methods:** KS (KetoForce^®^ (KetoSports, Frisco, TX, USA)) were tested in healthy subjects (11.7 g and 15.6 g βHB) and patients (15.6 g βHB 3×/day over 3 days). KE (KE4^®^ (KetoneAid, Falls Church, VA, USA)) containing 10.0 g βHB were applied in healthy subjects (once) and in patients (3×/day over 2 days). For the KD, KetoCal^®^ 2.5:1 LQ MCT MF Vanilla (Nutricia, Frankfurt, Germany) was applied via percutaneous endoscopic gastrostomy over four weeks. Regular capillary βHB measurements were conducted, and adverse events were recorded. **Results:** Between January 2021 and March 2025, we treated nine patients with ALS and two healthy subjects at the Department of Neurology of Ulm University, Germany. KE and KS increased βHB temporarily. However, the elevation was more pronounced following KE (maximum 2.2–2.7 mmol/L vs. 0.8–1.2 mmol/L). The KD increased βHB levels continuously with nighttime fluctuations. No adverse events occurred under KE. KS caused diarrhea in 3/5 patients and 1/2 healthy subjects. The KD was well tolerated, with mild gastrointestinal symptoms occurring in all patients. **Conclusions:** All ketogenic approaches increased βHB blood levels. While the KD and KE provided good tolerability, KS caused significant gastrointestinal side effects. KD seems to be an interesting candidate for future clinical studies, as it prompted a long-term increase in βHB while providing satisfying tolerability. Since maintaining a KD long-term is difficult for oral-feeding patients, KE may constitute a feasible alternative.

## 1. Introduction

Energy deficiency plays an essential role in the pathology of amyotrophic lateral sclerosis (ALS). Increased resting energy expenditure (REE) has been found in a large proportion of ALS patients and is associated with weight loss und shorter survival [1,2,3]. Mitochondrial dysfunction, especially impaired complex I of the respiratory chain, leads to an insufficient production of adenosine triphosphate (ATP), contributing to the energy deficit [4,5]. Furthermore, brains of ALS patients show impaired glucose utilization [6]. A study of 199 patients revealed that addressing the energy deficit therapeutically by applying a high-caloric, high-fat nutritional supplement (405 kcal/day) over 18 months induced a survival benefit in fast-progressing patients and reduced neurofilament light chain (NfL) serum levels compared to the placebo [7,8], confirming energy metabolism as a potential therapeutic target in ALS.

In terms of energy restriction, several physiological mechanisms ensure the maintenance of the energy supply by mobilizing available and alternative energy sources. Recent studies in ALS suggest that these mechanisms occur already in the early stages of disease, even when BMI is still normal [9]. Fatty acids are released from the adipose tissue and enter β-oxidation in mitochondria, resulting in the production of acetyl-CoA. Acetyl-CoA can then undergo the citrate cycle, which finally leads to the production of ATP. Alternatively, in case of a catabolic state, acetyl-CoA is metabolized to ketone bodies, particularly β-hydroxybutyrate (βHB). Unlike free fatty acids, βHB can cross the blood brain barrier. Moreover, studies have shown that more ATP is generated from βHB compared to glucose (10,500 g ATP/100 g βHB vs. 8700 g ATP/100 g glucose) [10,11,12]. Thus, βHB serves as a high-energetic substrate during periods of increased energy needs, fasting, or glucose restriction. βHB is also produced during sleep or extensive physical exercise [13]. In a previous study, βHB restored the function of complex I of the respiratory chain and consequently increased ATP production in cultured neurons, in which this complex had been pharmaceutically blocked [14].

βHB can be administered in two ways: first, exogenously in the form of ketone salts (KS) or ketone esters (KE), in addition to regular food intake, or, secondly, by applying a ketogenic diet (KD), which consists of high-fat nutrition with very restricted carbohydrate intake, thereby triggering the production of βHB. The KD is already a well-established and effective therapy in patients with therapy-refractory epilepsy [15,16]. The possible positive effects of a KD are also currently being discussed regarding other neurodegenerative diseases, such as Alzheimer’s and Parkinson’s disease [17,18]. Since the human brain depends on glucose and cannot metabolize fatty acids and since glucose metabolism is impaired in ALS [6], βHB may consequently serve as a beneficial additional and alternative energy source. In recent studies, KS and the KE provided acceptable tolerability in healthy subjects [19,20]. In the *SOD1* mouse model of ALS, KD resulted in a longer maintenance of motor function, higher body weight, higher preservation of motor neurons in the spinal cord, and higher ATP synthesis compared to control mice receiving standard nutrition [21].

However, the KD is difficult to implement due to strict carbohydrate restriction, which is further complicated in oral feeding ALS patients, who often have limited food choices due to dysphagia. Therefore, patients with percutaneous endoscopy gastrostomy (PEG) constitute an optimal study cohort as nutrition can be precisely controlled.

In this prospective case series, we aimed at investigating feasibility and tolerability of exogenous oral KS and KE, as well as a KD via PEG, in patients with ALS to identify the optimal intervention for a subsequent clinical trial investigating efficacy.

## 2. Materials and Methods

### 2.1. Study Population

Between January 2021 and March 2025, we treated nine patients with sporadic ALS and two healthy controls at the Department of Neurology of Ulm University, Ulm, Germany. Treatment was done in accordance with the Declaration of Helsinki, International Conference on Harmonization Guideline for Good Clinical Practice. It was approved by the independent ethics committee of Ulm University (approval numbers 145/21, 423/21, and 511/20). This case series contributed to the KETO-ALS (Efficacy and Tolerability of Beta Hydroxybutyrate Ester in Patients With Amyotrophic Lateral Sclerosis (ALS), clinicaltrials.gov NCT04820478, date of registration: 3 March 2021) and PEGASUS (Hypercaloric PEG Nutrition in ALS to Sustain Energy Homeostasis, clinicaltrials.gov NCT06877143, date of registration: 14 March 2025) trials regarding determination of the dosages that were used in these studies.

Eligible patients were required to have a diagnosis of ALS (possible, probable, or definite according to revised El Escorial Criteria [22]) and to give their written informed consent for treatment with one of three experimental ketogenic approaches described below. Patients receiving the KD were required to be fed via PEG. Patients did not have any metabolic or eating disorders prior to treatment.

Additionally, two healthy subjects were included to allow an evaluation of the effects of different dosages of both KS and KE on βHB levels and tolerability prior to treating patients.

### 2.2. Treatments

Three patients were treated with exogenous KS, two with KE, and four with the KD via PEG. Patients receiving KS were treated for three days, while those receiving KE were treated for two days. Healthy subjects received KetoForce^®^ (KetoSports, Frisco, TX, USA), a salt compound, containing 30 mL (11.7 g βHB, 55 kcal) or 40 mL (15.6 g βHB, 73 kcal) per serving. Patients received 40 mL (15.6 g βHB, 73 kcal) per serving. For the ester compound, we used KE4^®^ (KetoneAid, Falls Church, VA, USA), a ketone monoester consisting of D-βHB and R-1,3-butanediol, with 20 mL (10.0 g βHB, 48 kcal) per serving for both healthy subjects and patients. KS or KE were both administered three times daily. Patients receiving KS or KE were not instructed to follow a specific diet; it typically consisted of a standard Western European diet high in carbohydrates three times a day. Patients were instructed to follow their usual diet during and after treatment. During treatment with KS or KE, patients were instructed to eat three times daily, with at least a two-hour interval between meals and treatment. Adverse events were recorded during each capillary βHB measurement.

For the KD, we applied KetoCal^®^ 2.5:1 LQ MCT MF Vanilla (Nutricia, Frankfurt, Germany), covering 100% of individual calorie requirements, as determined by the sum of resting (REE) and physical energy expenditure (PEE). Indirect calorimetry was performed to determine REE. To calculate PEE, we used a modified version of the International physical activity questionnaire (IPAQ) [23]. Prior to PEG, patients usually had restricted food intake due to dysphagia. No patient followed a specific diet, which typically consisted of a standard Western European diet similar to those patients with KS and KE. After PEG insertion, there were no restrictions regarding additional oral food intake. However, due to dysphagia, the amount consumed was minimal (e. g. one yoghurt per day), except for one patient. This patient wished to continue oral nutrition in addition to PEG feeding, so we deducted those calories from the total calories supplied by the KD. Initially after PEG insertion, the ketogenic formula was mixed with standard tube nutrition. Then, we gradually increased the KD over the next few days to slowly acclimate metabolism to ketosis according to the following scheme: on the first day, patients received 100% of standard nutrition. On the second day, the KD and standard diet were mixed with a ratio of 1:1. On the third day, the KD was increased to 90% of the daily food intake. From day four, patients received exclusively the KD for a total of four weeks. The hospital observation period was between seven and 14 days for each patient. During the patients’ stay in hospital, patients were specifically asked daily about any side effects of the KD such as abdominal pain, nausea, and stool abnormalities. Additionally, routine blood sampling was performed daily to monitor for potential infections. Afterwards, patients were discharged and continued the KD at home. During this period, study participants were contacted by phone to collect data regarding tolerability and body weight. After this timeframe, patients could decide whether to continue KD or to switch back to standard tube feeding.

### 2.3. Outcome Parameters

Capillary ketone levels (βHB) were regularly measured after the intake of oral ketone bodies or during the administration of the KD using the On Call^®^ GK Dual ketone meter (Swiss point of care, IJsselstein, The Netherlands). In the KD, capillary βHB was monitored four times daily. For oral ketone bodies, measurements were taken every 10–15 min after intake with increasingly longer intervals as βHB decreased. Additionally, capillary glucose and blood pH values were monitored in patients receiving the KD to prevent relevant hypo- or hyperglycemia, as well as ketoacidosis. Tolerability and adverse events were systematically recorded.

## 3. Results

Baseline characteristics of individual study participants are displayed in Table 1.

### 3.1. Ketone Salts (KS)

**Figure 1:**
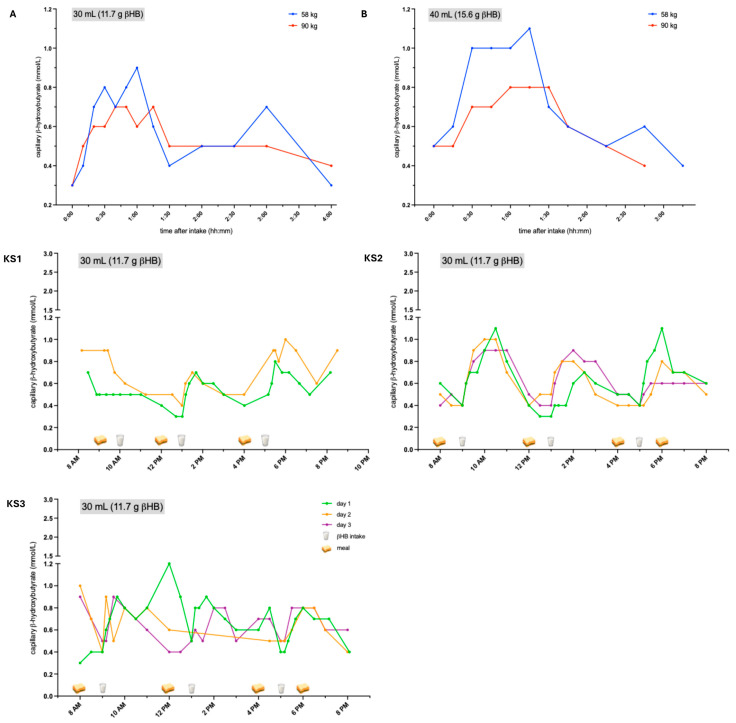
βHB levels after treatment with ketone salts. After KS administration, βHB levels increased to approximately 1.0 mmol/L two to three hours after intake, depending on the dosage and weight. A + B: healthy subjects. KS1–KS3: ALS patients. βHB: β-hydroxybutyrate, KS: ketone salts, ALS: amyotrophic lateral sclerosis.

In healthy subjects, KS increased βHB levels dosage- and body weight-dependently, with higher levels observed in subjects with lower body weights and after a higher dosage was applied (Figure 1, A + B). Patients treated with 30 mL (11.7 g βHB) of KS showed a peak increase in βHB up to approximately 1 mmol/L two to three hours after intake (Figure 1, KS1–KS3). In KS1, the elevation of βHB was only observed after the second and third intakes, probably since βHB levels were already relatively high in the morning. Due to diarrhea, flatulence, and nausea, KS1 discontinued treatment after two days. KS3 also experienced diarrhea; however, this patient was also receiving antibiotics due to PEG infection, which could have exacerbated the symptoms. This could also explain why this patient did not exhibit a regular pattern of βHB levels. KS2 did not experience any adverse events. Body weight (KS1 > KS2 > KS3) negatively correlated with βHB levels (KS3 > KS2 > KS1).

### 3.2. Ketone Esters (KE)

**Figure 2:**
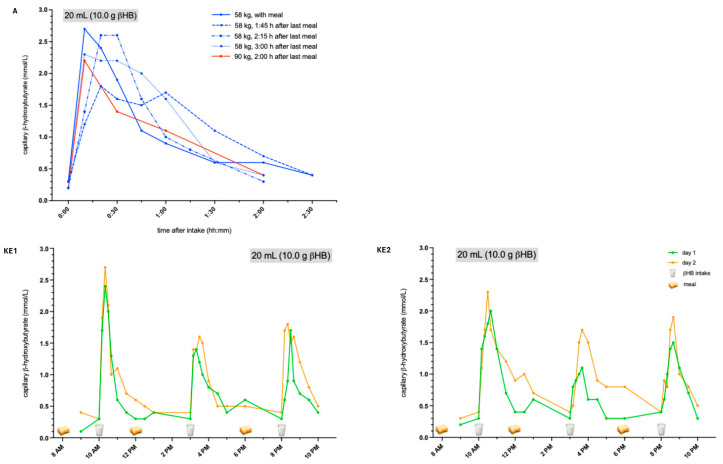
βHB levels after treatment with ketone esters. KE immediately increased βHB levels after one to two hours, reaching the highest levels at approximately 1.5 to 2.5 mmol/L. A: healthy subjects. KE1 + KE2: ALS patients. βHB: β-hydroxybutyrate, KE: ketone esters, ALS: amyotrophic lateral sclerosis.

Compared to KS, the βHB peak after the intake of KEs was achieved immediately after intake with a maximum of about 1.5 to 2.5 mmol/L (Figure 2, KE1 + KE2). This increase lasted for one to two hours and was therefore shorter compared to KS (two to three hours). Despite the significant difference in body weight between the two patients (37.5 kg vs. 67.5 kg), βHB levels were about similar. In healthy subjects, the increase in βHB following KE showed a weak negative correlation with body weight (Figure 2, A). Neither patients nor healthy subjects reported any gastrointestinal side effects. One healthy subject was tested with varying time intervals between the intake of KE and the preceding meal. This revealed a less pronounced increase in βHB when the last meal was consumed 1:45 h prior to KE, compared to longer time intervals or simultaneous intake. No significant differences were observed when KE were administered simultaneously with the meal, or after time intervals of 2:15 h, or 3:00 h.

### 3.3. Ketogenic Diet (KD) via PEG

**Figure 3:**
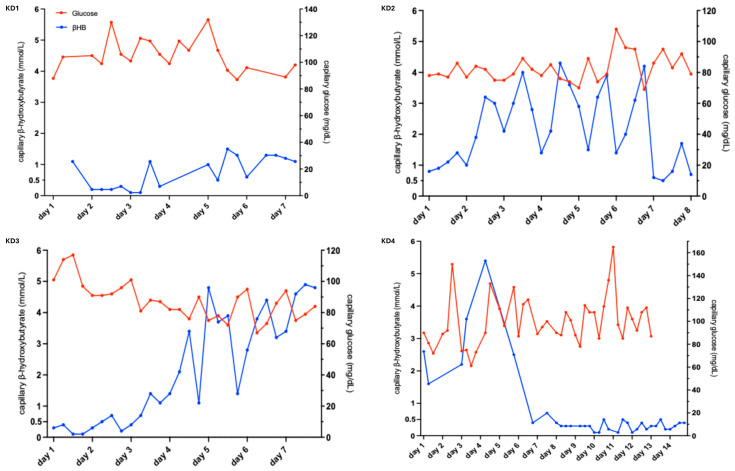
Individual βHB and glucose levels during the ketogenic diet. The KD prompted βHB levels of approximately 1.0 to 5.0 mmol/L, with higher levels during the day when applied correctly and in the absence of infection. KD1–KD4: ALS patients. βHB: β-hydroxybutyrate, KD: ketogenic diet, ALS: amyotrophic lateral sclerosis.

All four patients underwent PEG insertion immediately before starting the KD. In all four cases, PEG was indicated due to severe dysphagia and/or weight loss. KD2 and KD3 demonstrated progressive increases in βHB, respectively, which exceeded 1 mmol/L within the first two to three days (Figure 3, K2 + KD3). Subsequently, we observed fluctuations in βHB levels with lower levels at night and higher levels during the day, as the ketogenic diet was only applied during the day. Glucose measurements revealed no significant hypo- or hyperglycemia. KD1 exhibited lower βHB levels compared to the other patients (0.1–1.5 mmol/L vs. 0.5–4.3 mmol/L in KD2; 0.1–4.9 mmol/L in KD3; 0.1–5.4 mmol/L in KD4), most likely since KD1 consumed a large amount of additional nonketogenic oral nutrition (Figure 3, KD1). KD4 had higher βHB levels initially, but lower levels during the following days while experiencing diarrhea, which was most likely a side effect of the antibiotic treatment due to a PEG infection (Figure 3, KD4). pH levels were normal in all patients and did not change during the treatment at the clinic (Table 1).

Adverse events were primarily gastrointestinal problems (Table 1). Two of four patients initially experienced diarrhea, which stopped after a few days. Three patients reported flatulence. Two patients experienced a feeling of fullness. One patient suffered from abdominal pain and burping frequently. Among patients, who consumed little to no additional oral nutrition, side effects were mild gastrointestinal symptoms. In contrast, KD1 experienced a more severe feeling of fullness and flatulence, most likely because oral nutrition remained a major component of the diet. Therefore, this patient decided to stop the KD after 21 days due to difficulties in maintaining the amount of PEG nutrition. Since the reported gastrointestinal symptoms also commonly occur in patients after PEG insertion, it is difficult to determine whether these problems were caused by the procedure itself, the new type of nutrition, or malnutrition prior to PEG insertion. Based on our experience, the duration and intensity of side effects were similar to what we typically observe in patients after PEG insertion and the initiation of tube feeding. During the observation period, no patient discontinued treatment due to adverse events. KD3 died one day before the end of the observation period due to natural disease progression. One patient deliberately continued the KD after the observation period ended. Body weight remained relatively stable in all four patients (Table 1).

## 4. Discussion

In this prospective case series with nine ALS patients, we evaluated feasibility and tolerability of various ketogenic therapeutic approaches, including KS, KE, and the KD. Additionally, KE and different dosages of KS were tested in two healthy individuals.

We found that all approaches increased capillary βHB levels to varying degrees. Exogenous ketone bodies (both KS and KE) prompted a temporary increase in βHB over several hours. In contrast, the KD caused a persistent increase in βHB levels, which were higher during the day. In contrast to KS, which resulted in a smaller increase and a later peak of capillary βHB, KE prompted an immediate and more pronounced elevation. These findings are consistent with previous studies, in which exogenous KS or KE were tested in healthy subjects [19,24,25,26]. However, when comparing these different approaches, it must be kept in mind that, due to the nature of individual treatments, the observation period differed: 3 days for KS, 2 days for KE, and approximately 1 month for the KD. We also had no control group since this was a case series.

The case series on the KD for patients with ALS and PEG showed that ketosis was rapidly achieved within the first few days. Of note, all patients underwent PEG insertion shortly before establishing the KD. Therefore, it can be assumed that these patients had insufficient food intake prior to PEG insertion, which facilitated the achievement of ketosis. Importantly, βHB levels were influenced by the amount of additional non-ketogenic food consumed orally. This correlation must be considered when designing clinical studies in terms of inclusion and exclusion criteria, stratification, and statistical analysis. Levels of βHB were similar to those in a previous study investigating patients with brain injury who received the same intervention (Nutricia KetoCal^®^ 2.5:1) [27]. Even at night, when tube feeding was not administered, patients in our study had βHB levels above 1 mmol/L. Thus, as opposed to exogenous ketone bodies, ketosis can be maintained continuously by administering a KD.

Regarding adverse events, KS caused severe diarrhea in 2/3 patients and in 1/2 healthy subjects, which subsided after treatment ended. One patient prematurely discontinued treatment due to these symptoms. These side effects, most notably diarrhea, were also reported in previous studies [25,26] and are probably caused by the osmotic effects of potassium and sodium. Three of four patients receiving the KD also reported mild diarrhea during the first week. It is important to note that gastrointestinal side effects are also common with standard tube feeding during the first few weeks after PEG insertion. Since it is not possible to differentiate between side effects due to KD or PEG insertion, the reported side effects must be carefully interpreted. In contrast, previous studies investigating the KD in patients with pharmacoresistant epilepsy typically found constipation, while nausea was frequently found in both indications [15]. One reason for the relatively good tolerance of the KD in our patients may be its mild formulation with a ratio of 2.5:1 (fat: carbohydrates + protein). Conversely, patients with epilepsy, for whom the KD has been much more extensively investigated, typically receive a KD with a higher fat content (e.g., 3:1 or 4:1). In these patients, the severity of side effects has been found to correlate with an increasing fat-to-carbohydrates/protein ratio [15]. In contrast to KS and the KD, we did not observe any adverse events in patients treated with KE. This finding is also consistent with previous studies in healthy subjects [19,24].

In summary, patients with ALS can achieve short-term (KS and KE) or long-term (KD) ketosis by consuming oral ketone bodies as salt or ester compound or by following a KD. Due to the energy deficit found in most ALS patients that negatively influences disease progression and the impaired glucose metabolism associated with ALS, ketone bodies may serve as an effective alternative energy source. Since all therapeutic approaches aim to provide more energy, KS and KE must be administered in addition to other forms of caloric intake, rather than in place of them. For the KD, caloric needs can either be determined by using a standardized formula depending on sex, weight, and age (e.g., Harris-Benedict-formula), or by performing indirect calorimetry. Assuming that these ketogenic approaches can at least partially compensate for the energy deficit, they may potentially impact the rate of neurodegeneration and muscle atrophy. While exogenous ketone bodies temporarily increase βHB levels, the KD maintains continuous ketosis with nighttime fluctuations. Considering the promising results in the animal model [21], the KD might therefore constitute an interesting candidate for a clinical trial. Since PEG enables optimal control of nutritional intake, the KD administered via PEG appears to be an easily applicable nutritional intervention.

Since it is difficult to establish a KD in orally feeding patients due to strict carbohydrate restriction and dysphagia, exogenous ketone bodies may constitute a feasible alternative, although some of the potentially positive metabolic effects prompted by the KD are absent. In our study, we observed higher levels of βHB after the intake of KE compared to KS, which was also reported in previous studies [24]. Furthermore, we found that KE were better tolerated than KS, which frequently caused diarrhea [19,24,25,26]. Since gastrointestinal side effects might exacerbate weight loss, we would consider KS as inferior to KE for further clinical studies in ALS. However, due to the small sample size, we cannot draw a definitive conclusion about tolerability. Therefore, larger clinical studies with standardized recording of side effects over a longer time period are needed.

In our case series, we observed slightly higher βHB levels in leaner study participants compared to those with higher body weight following KS or KE ingestion. In healthy subjects, we observed slightly different responses to KE depending on whether they were fasting. Similarly, the aforementioned study by Stubbs et al. showed lower βHB levels in fed subjects compared to fasting subjects [24]. Another study found that, compared to subjects on a standard diet, athletes in a ketogenic state experienced twice the increase in βHB [28]. These results may suggest that the dosage of KE should be adjusted according to body weight. Furthermore, it may be beneficial to maintain an interval of several hours between meals and KE ingestion.

Assuming that providing additional and alternative energy substrates in the form of βHB counteracts the energy deficit and slows down disease progression in ALS, a KD may constitute an interesting candidate for further investigation. However, due to the difficulty of establishing the KD in oral-feeding ALS patients, exogenous ketone bodies may be a feasible alternative intervention in these individuals.

In summary, this case series demonstrated feasibility and adequate tolerability of different ketogenic interventional approaches in ALS, especially KE and a KD, paving the way for a subsequent clinical trial to investigate efficacy of these approaches.

## Figures and Tables

**Table 1:** Baseline characteristics of individual study participants. βHB: β-hydroxybutyrate, KD: ketogenic diet, KS: ketone salts, KE: ketone esters, HS: healthy subject, BMI: body mass index, f: female, m: male, PEG: percutaneous endoscopic gastrostomy.

Patient/Subject ID Sex	KD 1 (f)	KD 2 (m)	KD 3 (f)	KD 4 (m)	KS 1 (m)	KS 2 (m)	KS 3 (m)	KE 1 (f)	KE 2 (m)	HS 1 (f)	HS 2 (m)
**Height (cm)**	174	176	160	185	173	180	178	160	184	160	180
**Weight (kg)** **(prior to KD -> after 4 weeks)**	69.2 -> 68.5 (after 19 days)	66.0 -> 63.0	50.0 -> no follow-up	46.5 -> 48.5	70.7	56.8	51.0	37.5	67.5	58.0	90.0
**BMI (kg/m^2^)**	22.9	21.3	19.5	13.6	23.6	17.5	16.1	14.6	19.9	22.7	27.8
**Body composition** **fat mass** **fat free mass** **body cell mass**	26.8 kg (38.9%) 42.2 kg (61.1%) 18.4 kg (26.6%)	22.3 kg (34.8%) 41.7 kg (65.2%) 19.7 kg (30.7%)	18.6 kg (37.3%) 31.4 kg (62.8%) 8.6 kg (17.2%)	9.3 kg (19.3%) 38.8 kg (80.7%) 9.9 kg (20.6%)							
**Capillary βHB (mmol/L)**	0.1–1.5	0.5–4.3	0.1–4.9	0.1–5.4	0.3–1.0	0.3–1.1	0.3–1.2	0.1–2.7	0.2–2.3	KS: 0.3–1.1 KE: 0.2–2.7	KS: 0.3–0.8 KE: 0.2–2.2
**Calories/volume via PEG** **(for KD)**	1224 kcal 800 mL + oral calories	1836 kcal 1200 mL	1836 kcal 1200 mL	2448 kcal 1600 mL							
**Additional oral nutrition** **(for KD)**	++++	+	−	+							
**pH (for KD)** **mean (min–max)**	7.44 (7.41–7.47)	7.39 (7.36–7.41)	7.41 (7.36–7.48)	7.40 (7.36–7.42)							
**Adverse events**						none		none	none	KE: none	none
**Diarrhea**		+ (initially)	+ (initially)	+ (antibiotics)	++		++ (antibiotics)			KS: ++	
**Flatulence**	+		+	+	+						
**Feeling of fullness**	++	+									
**Loss of appetite**	++										
**Nausea**			+		+					KS: +	
**Abdominal pain**		+									
**Burping**		+									
**Outcome**	discontinued after 21 days		deceased one day before end of observation	continued after observation phase	discontinued after 2 days						

+, mild; ++, moderate; ++++, extensive.

## Data Availability

Individual participant data that underlie the results reported in this article, after de-identification (text, tables, and figures), will be available. Data will be available beginning 3 months and ending 5 years following article publication. Data will be shared with researchers who provide a methodologically sound proposal. Data will be shared for analyses to achieve the aims in the approved proposal. Proposals should be directed to christine.herrmann@uni-ulm.de; to gain access, data requestors will need to sign a data access agreement. Data are available for 5 years at https://www.uniklinik-ulm.de/neurologie.html (accessed on 18 May 2026).
